# Modulation of Tumor Tolerance in Primary Central Nervous System Malignancies

**DOI:** 10.1155/2012/937253

**Published:** 2012-01-24

**Authors:** Theodore S. Johnson, David H. Munn, Bernard L. Maria

**Affiliations:** ^1^Department of Pediatrics, Georgia Health Sciences University, 1120 Fifteenth Street, BT-1852, Augusta, GA 30912, USA; ^2^Immunotherapy Center, Georgia Health Sciences University, 1120 Fifteenth Street, CN-4141A, Augusta, GA 30912, USA; ^3^GHSU Cancer Center, Georgia Health Sciences University, 1120 Fifteenth Street, CN-4141A, Augusta, GA 30912, USA

## Abstract

Central nervous system tumors take advantage of the unique immunology of the CNS and develop exquisitely complex stromal networks that promote growth despite the presence of antigen-presenting cells and tumor-infiltrating lymphocytes. It is precisely this immunological paradox that is essential to the survival of the tumor. We review the evidence for functional CNS immune privilege and the impact it has on tumor tolerance. In this paper, we place an emphasis on the role of tumor-infiltrating myeloid cells in maintaining stromal and vascular quiescence, and we underscore the importance of indoleamine 2,3-dioxygenase activity as a myeloid-driven tumor tolerance mechanism. Much remains to be discovered regarding the tolerogenic mechanisms by which CNS tumors avoid immune clearance. Thus, it is an open question whether tumor tolerance in the brain is fundamentally different from that of peripheral sites of tumorigenesis or whether it simply stands as a particularly strong example of such tolerance.

## 1. Introduction

Central nervous system (CNS) tumors account for high rates of morbidity and mortality [1]. In children, CNS tumors represent the most common solid tumors with more than 3100 newly diagnosed patients in the United States annually [2]. Overall 5-year survival statistics are a dismal 35% in adult patients while they approach 75% in the pediatric population [1, 2], likely owing to fundamental differences in tumor biology. Even so, more children die each year from brain tumors—more than 2700 per year [2]—than from any other cancer. Patients with aggressive CNS tumors (glioblastoma multiforme, diffuse intrinsic pontine glioma, atypical teratoid/rhabdoid tumor, etc.) fare particularly poorly due to the high grade infiltrative nature of their disease and fundamental resistance to radiotherapy and current chemotherapy regimens. In fact, patients with high grade gliomas generally succumb to progression of persistent or recurrent disease [3].

 CNS tumors may have a specialized immune biology that allows evasion of immune clearance and promotion of tumor-growth, and the tissue milieu within which a CNS tumor naturally grows may be especially important to supporting this immunobiology. The term “immune privilege” has been used to describe deficient or defective adaptive immune responses that translate to an absence of tumor-specific immune responses (Table 1). Treatment of brain cancers is further complicated by the presence of a small molecule exclusion system, the blood-brain barrier, which limits the CNS penetration of many chemotherapeutics. Despite the complexity of this blood-brain barrier, however, it does not block lymphocytes or myeloid cells from migrating to sites of inflammation or tumor growth [4, 5].

 In fact, brain tumors contain large numbers of tumor associated macrophages (TAMs) and microglia as well as tumor infiltrating lymphocytes. These cellular components of the immune system apparently coexist with the developing tumor, and while antitumor responses are possible within the CNS [80], they are typically ineffective [38, 81–83]. In fact, the privileged status that brain tumors enjoy with respect to immune responses appears to be driven by highly active and dominant local immune suppression [38, 81, 83], as is the case with peripheral solid tumors [45]. However, in CNS tumors, we speculate that this local tumor-specific tolerance may be augmented by the specialized mechanisms of CNS privilege [28, 39, 84, 85]. Gaining a better understanding of these tolerogenic mechanisms is critically important to improving the survivability of malignant CNS tumors, which currently resist our most aggressive and multimodal therapeutic strategies.

## 2. Immune Privilege in the Central Nervous System

### 2.1. The Immune Privilege Paradigm

Uncontrolled immune responses in the brain are more dangerous than in any other location, and the central nervous system enjoys a distinctly different immunology than peripheral tissues [29, 34, 35]. Classical CNS “privilege” was described phenomenologically in terms of diminished or absent immune responses [29, 35]; particularly compelling was Medawar's observation that tissue graft rejection was impaired in the brain [86]. Additional findings suggesting a unique immunology existed in the CNS included lack of lymphatic vessels and lymph nodes within the CNS [29], lack of dendritic cells resident within the brain parenchyma [29], low major-histocompatibility (MHC) expression levels on all cells within the CNS—including low MHC-II on resident microglia [29, 35], and widespread presence of soluble anti-inflammatory mediators, such as vasoactive intestinal peptide [35], alpha melanocyte stimulating hormone [35], and transforming growth factor-beta (TGF-*β*) [35, 38]. Furthermore, production of inflammatory cytokines and nitric oxide by CNS resident myeloid cells, including macrophages and microglia, is suppressed by a cell-contact mediated receptor ligation to CD200, a ligand expressed by brain parenchymal cells [35]. Thus, the character and strength of immune responses in the CNS are fundamentally different than in the periphery. Presumably, these strict regulatory mechanisms [42] have evolved to preserve the nonrepairable brain tissue and avoid unchecked inflammation in a closed space that could otherwise lead to increased intracranial pressure, herniation, and death [29].

### 2.2. Leukocyte Entry into the Central Nervous System

Naïve T cells are effectively excluded from brain parenchyma by the tight junctions of the blood-brain barrier [28]. Thus, leukocyte trafficking generally occurs at very low frequency in quiescent brain [35]. Nonetheless, all the elements of an effective immune response—including dendritic cells, macrophages, and T cell lineages—can and do traverse the blood-brain barrier in inflammatory states [4, 29, 34, 35]. T cells usually become activated in extra-CNS sites, where they encounter an appropriate antigen before migrating into the CNS itself [29, 35]. T cells expressing the chemokine receptor CCR7 home effectively to the CNS via chemokine-mediated (CCL19 and/or CCL21) homing [29]. Leukocytes thus recruited enter the CNS at postcapillary venules by the standard process of tethering, leukocyte rolling, chemokine activation, adhesion, and diapedesis [29, 34, 38]. However, in the CNS, diapedesis appears to occur via transendothelial extravasation, rather than a paracellular route, which leaves the blood-brain barrier endothelial tight junctions intact [29, 85]. Once they have transmigrated through the vascular endothelium, these leukocytes find themselves in an enlarged perivascular space, the Virchow-Robin space [29, 34]. It is within this space that they will either encounter antigen to maintain their activated state, or fail to do so and die. To reach the CNS parenchyma, leukocytes still need to cross the *glia limitans* which is defined by the interlocking perivascular astrocyte foot processes [34]. Once in the CNS, however, activated T cells are free to carry out their effector functions [35].

### 2.3. Antigen Presentation in the Central Nervous System

A unique anatomical facet of CNS immunology is the lack of local draining lymph nodes. In fact, animal experiments have shown that labeled dendritic cells injected directly into CNS parenchyma do not appear to migrate from the site of inoculation [30], whereas dendritic cells (DCs) in the interstitial fluid of the CNS behave more like DCs in peripheral sites and are able to migrate to the cervical lymph nodes via perivascular channels [31]. Other studies have shown that rat dendritic cells and microglia injected into the striatum migrate to the perivascular space and exit through the vasculature to reach distant sites, such as spleen and mesenteric lymph nodes [32]. In contrast, dendritic cells injected into the cerebral spinal fluid (CSF) migrate to the B cell follicles of cervical lymph nodes [30], and they do so by traversing the cribriform plate to reach the nasal lymphatics [31]. This is similar to experiments in which radio-labeled protein infused into the CSF preferentially drains to deep cervical lymph nodes via the cribriform plate [28, 33]. Thus, the afferent arm of local CNS immune surveillance is quite complex and, in some contexts, may bypass traditional lymphatic routes of antigenic sampling.

 Not only do antigen-presenting cells (APCs) in the brain often fail to migrate into lymph nodes, but the CNS is also the only tissue with microglia as antigen-presenting cells, which imparts a unique immune biology to CNS-directed responses [36]. Microglia are derived from early monocytic cells during embryonic development [34, 37]. In adults and children, they can be replenished from progenitor cells in the CNS that have proliferative capacity for microglial renewal [34]. Microglia resemble resident perivascular macrophages with similar phenotypic markers and functional profiles [37]. Although resting microglia have a quiescent phenotype with low expression of MHC and costimulatory molecules, they have very dynamic motility, presumably consistent with their antigen-surveillance function [36]. In fact, effective responses to viral encephalitis depend upon microglial cytokine-mediated macrophage recruitment [34]. This cytokine production can lead to capillary leak and compromise the integrity of the blood-brain barrier [34], but such a breach will also cause local microglial activation and recruitment of circulating immune cells [36]. Thus, microglia play an important regulatory role in initiating responses to CNS infection and in modulating and directing intracranial immune responses.

## 3. Immune Privilege in the Setting of Central Nervous System Malignancy

### 3.1. General Events in Tumor Formation, Growth, and Survival

Tumors must develop complex stromal networks that promote vigorous growth but suppress adaptive immune responses—and tumors must accomplish this despite the presence of many intratumoral innate immune cells and tumor infiltrating lymphocytes [87, 88]. The stromal content of solid tumors is very large [89] (sometimes more stromal cells than tumor cells) and the paradoxical ability of this stroma to support growth yet suppress immune rejection is essential to the survival of the tumor.

#### 3.1.1. Important Factors in Oncogenesis

Malignant transformation occurs when a critical mass of genomic and epigenetic mutations leads to uncontrolled cell division, either dominated by a loss of cell cycle control [90–93] or by a defect in apoptotic pathways [92, 94–96]. This results in a cluster of neoplastic cells, derived from a single progenitor, which grow without the constraint of normal anatomical or tissue-specific limitations. These changes often coincide with a dedifferentiated phenotype that may be a distinct consequence of the underlying genetic defects. At this early stage, potentially immunogenic tumor-associated shared “self” antigens [97] and truly foreign neoantigens [97, 98] first appear as epitopes found within proteins derived from mutated or dysregulated genes. Thus, in order to become established, grow, and progress, CNS cancers must evade the immune system even at this early stage.

#### 3.1.2. Important Events in Tumorigenesis

**Figure 1:**
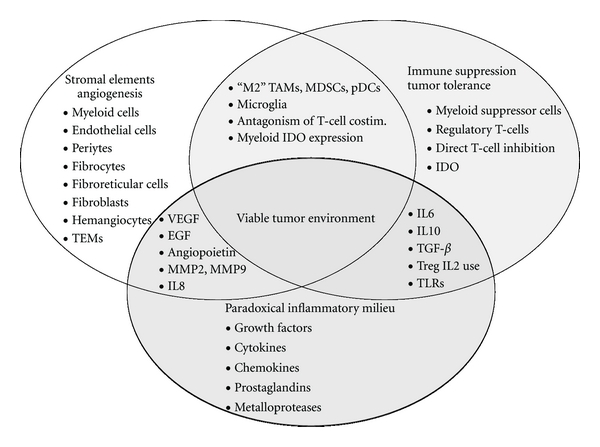
Viable tumor environment. Tumor survival is dependent upon an exquisite interplay between the critical functions of stromal development and angiogenesis, local immune suppression and tumor tolerance, and paradoxical inflammation. TEMs: TIE-2 expressing monocytes; “M2” TAMs: tolerogenic tumor-associated macrophages; MDSCs: myeloid-derived suppressor cells; pDCs: plasmacytoid dendritic cells; co-stim.: co-stimulation; IDO: indoleamine 2,3-dioxygenase; VEGF: vascular endothelial growth factor; EGF: epidermal growth factor; MMP: matrix metaloprotease; IL: interleukin; TGF-*β*: transforming growth factor-beta; TLRs: toll-like receptors.

Tumorigenesis is the process by which nascent oncogenic cell clusters transform into a viable tissue environment with a secure vascular supply and robust stromal elements capable of supporting the rapid and sustained tumor tissue growth. This transformation involves a complex series of events. Firstly, stromal elements must be recruited and developed into a subtumoral compartment that serves as a scaffold and provides crucial growth factors leading to angiogenesis and tumor tissue maintenance [56–58, 67–69, 71, 99, 100]. This stroma must be capable of supporting and promoting a dominant local immune suppression that leads ultimately to crucial tumor tolerance [11, 43, 62, 87, 101–106]. Furthermore, this tolerance, and the stroma that supports it, is characterized by a paradoxical inflammatory milieu that consists of chronic, low-grade, specialized inflammation, which we speculate may drive a characteristic “tissue remodeling” program that is normally meant for sterile wound healing, and which is actively suppressive for *de novo* T-cell responses within that milieu [59, 67–69, 71, 74, 88, 99, 107–110]. Thus, tumor survival is dependent upon these closely related and complimentary mechanisms: stromal formation and angiogenesis, immune suppression leading to the establishment of tolerance, and maintenance of both of these by a paradoxical inflammatory program usually reserved for sterile wound healing (Figure 1).

#### 3.1.3. Stromal Formation and Angiogenesis

Development of vascular access for nutrient delivery is essential for early cancer cell clusters to develop into a tumor capable of further growth. Thus, the developing tumor must attract primitive stromal elements that can provide the foundation for tumor vascularization. This requirement defines the tumor microenvironment as an inflammatory tissue environment where chemokines [67, 68, 71, 88, 100, 108], cytokines [59, 67–69, 71, 74], and various growth factors [67, 68, 74, 87, 107, 109, 110] provide critical signals for migrating stromal elements—myeloid cells, vascular and lymphatic endothelial cells, pericytes, fibrocytes, fibroblasts, fibroblastic reticular cells, and so forth—to take up residence and functionally support tumorigenesis in the periphery. Once present, many of these stromal cell types, such as vascular [74] and lymphatic [75] endothelial cells, can engage in proliferation and can, themselves, secrete chemokines, cytokines, and growth factors to support the everincreasing stromal needs of the growing tumor [74, 75]. Thus, the sterile inflammation of the tumor microenvironment recruits a complex stromal network to promote tumor growth and tissue remodeling as necessary. In fact, tumor tissue remodeling is necessary for the initiation of angiogenesis and occurs in a dynamic fashion [74], with a downregulation of antiangiogenic secreted proteases, such as ADAMTS-8 in brain tumors [107], and increased secretion of proangiogenic matrix metalproteases (MMPs), such as MMP2 and MMP9 [58, 67].

 Many tumors, including gliomas, are capable of secreting other growth factors, such as vascular endothelial growth factor (VEGF) [72, 91], TGF-*β* [56–59], and progranulin [109, 110]. However, the intricate and crucial process of angiogenesis is mediated largely by CNS tumor infiltrating macrophages [67, 68] and microglia [56–58]. In fact, several tumor-associated macrophage subsets directly promote angiogenesis. Tumor-associated macrophages of the tolerogenic “M2” phenotype drive angiogenesis by secreting VEGF, MMP9, epidermal growth factor (EGF), and interleukin-8 (IL8) [67, 71]. Although their exact role in angiogenesis remains to be elucidated, Tie-2 expressing monocytes (TEMs) inhabit perivascular areas where Tie-2 serves as the receptor for angiopoietins [67, 68, 71]. Other, more heterogeneous myeloid populations involved in angiogenesis include hemangiocytes described as expressing CXCR4, VEGF receptor-1, Tie-2, Sca-1, and CD117 [67, 68] and myeloid-derived suppressor cells (MDSCs) which express CD11b, Gr1, and CXCR4 [67–69]. In CNS tumors, resident brain microglial cells migrate into the developing tumor in response to the same chemotactic signals that attract the myeloid subsets [39]. Although not as well studied, it is clear that microglia also contribute to angiogenesis by secreting VEGF, EGF, TGF-*β*, and MMP9 [57, 72].

### 3.2. Functional versus Anatomical CNS Privilege

As noted above, inflammatory responses in the CNS are more tightly regulated than other sites [29, 34, 35, 38, 86]. Historically, it has been assumed that much of this reduction in immune responses was a passive anatomical phenomenon, resulting from the lack of effective antigen-presenting cells and lymphatic drainage, combined with anatomic exclusion of circulating lymphocytes by the blood-brain barrier. Given this, it was natural to assume that CNS tumors partook of a similar, anatomically-based protection due to their “privileged” location [28, 111]. Such mechanisms doubtlessly play a role, but a new paradigm is also emerging, in which CNS tumors also exploit mechanisms of active immune suppression—both natural suppressive mechanisms that exist within the CNS and pathologic immunosuppressive mechanisms induced by the tumor. Together, these mechanisms allow tumors to actively protect themselves from immune clearance [28, 42, 111]. The need for active immune suppression becomes logical when we remember that the presence of the tumor itself often disrupts many of the passive anatomic barriers in the CNS, for example, by altering the blood-brain barrier in the tumor vasculature, enhancing leukocyte trafficking, creating chronic inflammation, and introducing new populations of antigen-presenting cells inside the tumor. Thus, tumors in the CNS are not “invisible” to the immune system, and tumors must actively suppress immune responses against themselves in order to survive. The importance of understanding these active mechanisms of suppression lies in the fact that active mechanisms represent attractive therapeutic targets if they can be disrupted.

### 3.3. Local Immune Suppression and Establishment of Tumor Tolerance

As tumor size increases, tumor cell turnover also increases—and so does the volume of tumor-derived antigens. Many of the tumor-associated “shared-self” antigens could potentially be recognized by the immune system, because they may be excluded from central tolerance by virtue of their cellular, anatomical, or developmental expression patterns [97]. In the case of authentic tumor-specific neoantigens, which are derived from the protein products of mutated genes [97, 98], the immune system by definition has never acquired central tolerance. Despite this, however, the immune system behaves as if it were tolerant to tumor-derived antigens, whether shared “self” or neoantigens. One possible hypothesis to explain the lack of immune response in the presence of large amounts of these potential immunogens is that, analogous to the processes important in maintaining adaptive immune tolerance to normal tissues undergoing rapid cell turnover [112], antigens may be processed locally in a manner that avoids systemic immune activation. It appears that the type of APC that processes these antigens is critically important to the outcome—tolerance versus stimulation [113]—but the specific molecular mechanisms by which tolerance is created remain unclear. Nevertheless, the result can be dramatic: in one murine spontaneous-tumor model in which every tumor cell carries a potently immunogenic xenoantigen, the immune system still invariably becomes tolerant to the xenoantigen unless the host is vaccinated against the xenoantigen *prior* to tumorigenesis [102].

#### 3.3.1. General Issues Regarding Suppression of Antitumor Immunity

Autochthonous peripheral tumor models suggest that tumor-specific tolerance may become established very early in tumorigenesis, as observed in mouse models of pancreatic ductal adenocarcinoma [101], 4T1 mammary tumors [43], B16F10 melanoma [43, 45], and AB1 mesothelioma [43]. This observation can be explained conceptually, in part, by the cancer immunoediting hypothesis in which an initially effective antitumor response “edits” the tumor cell repertoire by removing any cells that are immunogenic [62, 104]. Thus, in this model, the early interactions between immune cells and tumor cells actively select for later immune suppression by favoring tumor cells capable of escaping immune clearance.

 Some of these escape mechanisms are passive. Passive tumor escape mechanisms were the first to be discovered and explored, and these include the emergence of tumor cell antigen-loss variants, downregulation of MHC-I expression, impairment of antigen processing or MHC binding in tumor cells, and suboptimal costimulatory molecule expression on tumor cells [62]. However, more recently, a variety of active immune suppressive mechanisms have been identified, which lead to dominant and profound tumor-induced tolerance (Table 1). These active mechanisms include secretion of soluble immune-modulating factors by tumor cells themselves, direct suppression of lymphocyte activation or effector function, and recruitment of myeloid or lymphoid suppressor cells. Immunosuppressive cytokines and growth factors known to be secreted directly by tumor cells include IL6 [114], IL10 [60], TGF-*β* [56, 58, 59, 62, 115], and VEGF [62]. These soluble mediators may directly inhibit T cell activation, and this effect may be augmented by contact-mediated antagonism of T cell costimulatory pathways through ligation of cytotoxic T lymphocyte antigen-4 (CTLA-4) [11, 62] or the programmed death-1 (PD-1) receptor on T cells [11, 46, 62]. In fact, one of the PD-1 ligands, PD-L1, is upregulated by gliomas when the *PTEN* tumor suppressor gene is defective [66].

 Tumors and their stromal components are known to actively recruit regulatory immune cell subsets [87, 88, 101, 106, 116], especially regulatory T cells (Tregs) [43, 71, 88, 98, 99, 105, 116]. Tregs exert direct suppressive effects upon CD4 T cells and CD8 T cells via secretion of suppressive cytokines (IL10 and TGF-*β*)*;* consumption of IL2 in the local microenvironment (which deprives effector T cells of this critical growth factor); contact-mediated inactivation of antigen-presenting cells; induction of the immunosuppressive enzyme indoleamine 2,3-dioxygenase (IDO) [7, 43, 71], which is discussed below. In addition, activated Tregs within the tumor microenvironment can polarize tumor-associated macrophages toward the “M2” suppressive phenotype [71]. It is clear that Tregs play an important role in many tumors, although the degree to which different tumors depend on Tregs probably varies with context.

 Despite active suppression of adaptive immune responses, most tumors appear to have an inflammatory milieu resembling a state of chronic sterile wound healing [67, 68, 99, 117, 118]. This characteristic tumor-associated inflammation is critical for maintenance of stromal integrity [99], promotion of angiogenesis [74], and continued tumor tolerance [88, 99, 108]. While a wide variety of stromal elements contribute to the formation of this specialized environment, tumor cells [99, 118] and tumor-associated macrophages [67, 68, 74, 100, 117] secrete many of the key inflammatory mediators, including the growth factors, cytokines, chemokines, prostaglandins, and metaloproteases described above. Elaboration of these crucial factors may be driven by transcriptional activation caused by oncogenic mutations [119], toll-like receptor (TLR) signal transduction [99, 118], and/or cytokine- or growth factor-mediated signaling [74, 99]. Particularly important to the immune tolerogenic properties of the tumor are the effects of TGF-*β* secretion [59], including suppression of T-cell adaptive and natural killer (NK) cell innate antitumor responses, recruitment of suppressive myeloid cell subsets such as suppressive dendritic cells, TAMs, and MDSCs, and recruitment of regulatory T cell activity [7, 12, 59, 73]. As noted above, vascular [120] and lymphatic [75] endothelium may contribute to this inflammatory milieu with growth factors and chemokines, and it is widely appreciated that tolerogenic “M2” phenotype TAMs support angiogenesis by secreting VEGF, EGF, MMP9, and so forth [67, 71].

#### 3.3.2. Infiltrating Tumor-Associated Macrophages and Microglia Maintain the Stromal Microenvironment and Suppress T-Cell Responses in CNS Malignancy

**Figure 2:**
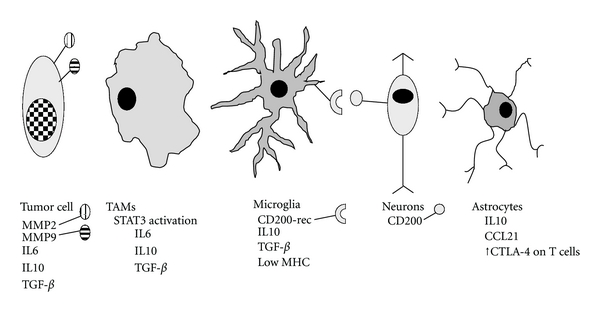
Tumor cells and stromal elements with immune suppressive functions. CNS tumor cells, especially glioma cells, may develop the ability to secrete cytokines including IL6, IL10, and TGF-*β* and can take advantage of membrane integrin-bound metaloproteases (MMP2 and MMP9) to facilitate motility and invasiveness. Tumor-associated macrophages (TAMs) bind IL6 and IL10 via their respective receptors, leading to phosphorylation and activation of STAT3, a transcription factor that upregulates TAM IL6, IL10, and TGF-*β* production and secretion. Ligation of the CD200 receptor on microglia by the ligand found on parenchymal neurons downregulates inflammatory cytokine and nitric oxide production by microglial cells. Microglial cells also have low expression of MHC-II and secrete IL10 and TGF-*β*. Astrocytes excrete IL10, and also CCL21, thus recruiting activated T cells which are then educated to upregulate CTLA-4 to antagonize costimulatory signals. IL10 promotes CNS tumor growth and migration, whereas TGF-*β* is an important regulator of tumorigenesis, angiogenesis, and tumor cell motility and invasiveness.

Tumor-associated macrophages (TAMs) and microglia are important for glioma tumor survival (Figure 2), as shown by the fact that ablation of these cells reduces tumor growth and improves survival in a murine syngeneic orthotopic glioma model [83]. TAMs process large volumes of dead and dying tumor cells without inciting adaptive immune responses, despite an apparently activated phenotype [67, 68, 71]. Tumor cell turnover is rapid in most solid tumors, and often the tumor core is devitalized as a result of central vascular insufficiency. This immense flux of cellular debris must be disposed of; a task that is largely borne by the tumor associated macrophages (but not, in the case of CNS tumors, by microglia) [39, 83]. This role for macrophages in tumors is reminiscent of other tolerogenic macrophage populations, for example, marginal zone macrophages which clear large amounts of apoptotic debris from the splenic circulation daily; tingible-body macrophages in germinal centers; Kupffer cells which process antigens from the portal circulation [112]. In none of these cases do macrophages provoke a pathological immune response to antigens from the dying cells that they ingest [112].

The classic trigger for inflammation is infection, in which activated APCs drive robust lymphocyte responses leading to pathogen clearance (albeit at the expense of local bystander tissue damage). However, as described above, certain inflammatory mediators are also critical to tumor establishment, growth, progression, and metastasis [68, 83, 117, 121, 122]. These occur in relatively tolerogenic tissue environments where adaptive immune responses against tumor-derived antigens have been blunted [100, 123]. This illustrates the point that the ultimate effects of inflammatory mediators depend not only on the character of the inflammation itself (e.g., sterile wound-healing tissue-remodeling type versus microbial pathogenic immune stimulation type) [99, 117, 118], but also upon the context in which it occurs (e.g., the actively immunosuppressive environment of tumors versus the stimulatory environment of infected tissue) [31, 35, 100, 123].

 Outside the CNS, it is known that stromal elements in tumors can contribute to tumor tolerance. In addition to the role of macrophages described above, mesenchymal cells and fibrocytes that express fibroblast activation protein can drive tumor tolerance independently of TAMs [87]. In addition, both stromal cancer-associated fibroblasts within the tumor and mesenchymal fibroblastic reticular cells and lymphatic endothelial cells in the tumor-draining lymph nodes are capable of secreting CCL21 [108, 124], which has been shown to attract CCR7-expressing tolerogenic cell populations (including Tregs and IDO-expressing cells) [88]. In the specialized environment of the CNS, these and other stromal cell subsets are normally excluded, and astrocytes perform many of the comparable stromal functions. As mentioned above, CNS tumors often disrupt the normal architecture of the brain, so some of the stroma in brain tumors may be ectopic, and resemble stroma in peripheral locations. However, astrocytes also have the ability to suppress T cell responses—both directly via upregulation of CTLA-4 expression [40] and by recruitment of regulatory T cells [125]. Also, CCL21 is secreted by glioma cells and tumor stromal cells and has been shown to directly promote glioma cell growth *in vitro* [83]. Astrocytes may therefore play a role in stromal-mediated tumor tolerance in the CNS.

 Glioma-derived tumor cells are capable of directly secreting immunosuppressive cytokines [35, 39], including IL6 [114], IL10 [60], and TGF-*β* [56, 115], and microglia and astrocytes have also been documented as sources of cytokines [57, 60]. Serum IL10 levels are elevated in patients with high-grade glioma, and IL10 enhances glioma cell growth and migration *in vitro *[60]. Although high levels of TGF-*β* can inhibit glioma cell growth *in vitro* [115], *in vivo* TGF-*β* plays a role in glioma tumorigenesis, angiogenesis, cellular motility, and invasiveness [56, 57, 115]. This characteristic enhancement of invasive potential is mediated by increased secretion and integrin-mediated glioma cell surface binding of MMP2 and MMP9 [56, 58]. Cytokines not only affect the tumor cells, but they also affect the neighboring tumor-associated macrophages as well. Cytokines such as IL6 and IL10 bind their respective receptors on the cell surface of TAMs, leading to phosphorylation and dimerization of signal transducer and activator of transcription-3 (STAT3). An autocrine loop is thus established, whereby additional IL6 and IL10 are produced as a result of their own signal transduction by TAMs, which also begin to secrete TGF-*β* as a result of phospho-STAT3 transcriptional activation [13, 61, 121]. Thus, a mutually reinforcing interplay may exist between stromal cell- and glioma-derived immune suppressive cytokines, the stromal cells (macrophages, astrocytes, and microglia), and the glioma cells themselves whereby tumor-related growth, invasiveness, and immunosuppression are regulated.

#### 3.3.3. Tumor Tolerance Mediated by Indoleamine 2,3-Dioxygenase

Indoleamine 2,3-dioxygenase (IDO) is an intracellular enzyme, involved in tryptophan catabolism, which is expressed by several murine and human APC subsets that engage in suppression of T-cell responses [14–24, 45, 47–49, 126–129]. IDO enzymatic activity degrades tryptophan via oxidative cleavage of the pyrrole ring, which results in production of kynurenine as well as other downstream metabolic products, including picolinic acid, quinolinic acid, and 3-hydroxyanthranilic acid [14, 130, 131]. IDO expression by specialized plasmacytoid dendritic cells in tumor-draining lymph nodes directly suppresses local tumor-specific T cell responses in the periphery and promotes activation of regulatory T cells [16, 20–22, 47]. Direct T cell suppression via IDO-expressing APCs occurs through activation of the general control nonrepressed-2 (GCN2) kinase pathway in T cells which are attempting to activate in the context of insufficient tryptophan stores [132]. GCN2 kinase is part of an integrated stress response pathway that senses uncharged tRNA and leads to abortive T cell activation. Recent work has also implicated the downstream tryptophan catabolites themselves in suppressing T cell responses by tumor-infiltrating lymphocytes and in experimental models of autoimmune encephalitis [130, 131].

 In a number of peripheral tumor models, IDO appears to function as a pivotal regulator of tolerance in the tumor-draining lymph node. IDO is expressed by specialized dendritic cells and other myeloid cells that potently suppress T cell responses [16, 18, 133]. Furthermore, IDO expression by dendritic cells in tumor-draining lymph node is necessary for certain forms of tumor-induced tolerance, a phenomenon which occurs in part via recruitment and induction of existing and new regulatory T cells [48]. Dendritic cells have been shown to drive T cell tolerance via the IDO pathway in both human [16] and murine [18] systems. Studies in mouse melanoma have shown that IDO expression by dendritic cells in draining lymph nodes suppresses CD8 T cell responses and leads to systemic tumor tolerance within just a few days [45, 48]. IDO can be induced by type I and type II interferons, by activated Tregs via CTLA-4 induced ligation of dendritic cell B7 molecules [22] and by STAT3-dependent mechanisms [134, 135].

**Figure 3:**
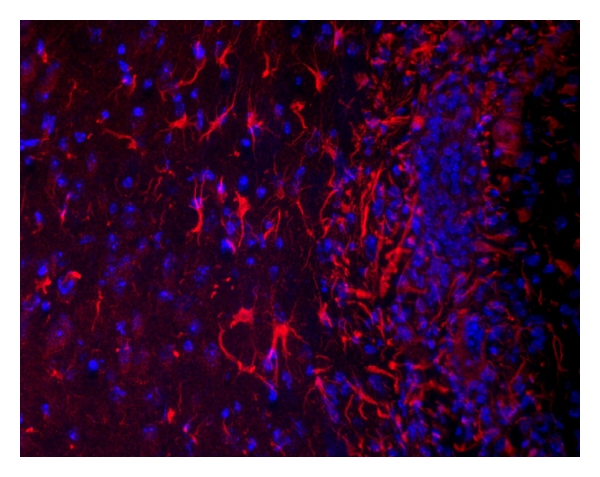
IDO-expressing astrocytes at the margin of a murine intracerebral GL261 glioma. IDO (red); nuclear counterstain (blue).

 Most of the preceding studies focused on dendritic cells, which are notably lacking in CNS tumors. Less is known about the role of IDO in TAMs and tumor-associated glial and microglial cells. Several lines of evidence suggest that IDO may play a role in suppressing CNS tumor-specific immune responses. Using immunohistochemistry, Uyttenhove demonstrated widespread IDO expression in nine of ten human glioblastoma biopsies. [50]. Human glioma cells upregulate IDO expression and enzymatic activity in response to Interferon-*γ* (IFN-*γ*) treatment *in vitro* [136]. IDO expression also can be induced by IFN*γ* in astrocytes, microglia, and perivascular macrophages both *in vitro* and *in vivo* as the result of CNS inflammation [41]. Furthermore, intense IDO expression is seen in astrocytes within a reactive gliosis at the margin of orthotopic murine glioma tumors (Figure 3), and IDO activity has been documented in TAMs from a rat orthotopic glioblastoma model using an immunohistochemical method to stain tissue for quinolinic acid, a downstream tryptophan metabolite [137]. Thus, IDO is expressed by many CNS tumors and their associated stroma, but mechanistic studies are needed to elucidate the immunologic role of this IDO expression.

### 3.4. Leukocyte Trafficking and Maintenance of Quiescent Vascular Endothelium within CNS Tumors

Leukocyte entry into the CNS is tightly regulated and appears to occur only by transmigration across the endothelium of post-capillary venules in the choroid plexus, meninges, and CNS parenchyma [38]. Thus, the most direct route for activated T cells to reach target tumor cells is transmigration across vascular endothelium within the tumor itself. This process is initiated via interactions between vascular cell adhesion molecule-1 (VCAM-1) and intercellular adhesion molecule-1 (ICAM-1) on endothelial cells and *α*_4_ *β*_1_-integrin and leukocyte function-associated antigen-1 (LFA-1), respectively, on T cells [29, 38, 84, 85]. In peripheral tumors, routine leukocyte margination within the tumor vasculature is hampered by diminished adhesion to the vessel walls, and this is thought to be the result of decreased endothelial adhesion molecule expression [76]. Chemo-attractant mediators may play an important role in activating the endothelial compartment so that it can support leukocyte binding for transmigration. Furthermore, leukocytes bearing chemokine receptors, including CCR1, CCR2, CCR7, and CXCR3, have been described in various models of CNS inflammatory disease and malignancy [29, 38]. Thus, the tumor may further shield itself from immune clearance by controlling the nature and quantity of cytokine and chemokine secretion by stromal elements, including antigen-presenting cells.

 While endothelial quiescence is an important mechanism whereby leukocyte trafficking into CNS tumors is minimized, CNS tumor vasculature is nonetheless profoundly aberrant, with significant downregulation of endothelial adhesion molecules [38, 76]. In addition, there is considerable crosstalk between the stromal and endothelial compartments, which is only complicated by contributions of the tumor cells and marginated immune cells to the inflammatory milieu. Furthermore, effective angiogenesis must occur for tumor survival, and this process is mediated both via VEGF secretion by tumor and stromal cells [38, 39] and by secretion of angiopoietins which sustain and augment the vasculogenic process by binding the receptor tyrosine kinase Tie-2, expressed by endothelial lineage cells [67, 68, 71, 77–79]. In an intriguing departure from lineage specificity, glioma-derived stem cells can engage in vasculogenic mimicry, giving rise to aberrant intratumoral vascular endothelium [138–143], which has been shown to be both radioresistant [140] and chemoresistant [141].

## 4. Therapeutic Strategies to Break Immune Privilege

### 4.1. Vaccination against Brain Tumor-Specific Antigens

In the face of the profound tumor-induced tolerance driven by the mechanisms detailed above, it is not surprising that attempts to develop vaccination-based immunotherapy have been met with difficulty. In murine brain-tumor models, vaccines can create early signs of immune responsiveness (microglial upregulation of MHC, reactive gliosis, and lymphocytic infiltration), but fail to produce tumor rejection [80]. More intensive immunotherapy, combining peptide-pulsed dendritic cell vaccination with tumor-specific T cell adoptive transfer, showed that tumor-specific T cells do migrate into the brain tumor resulting in smaller tumors with prolonged survival [144]. However, these regimens were demanding, requiring sublethal irradiation prior to T cell transfer and dendritic cell vaccine as well as IL2 cytokine therapy afterwards.

 Clinically, several very promising vaccines have been developed to target antigens on brain tumors [28]. Unfortunately, vaccination strategies against human glioblastoma have proven disappointing when used as single-agent therapy. Despite generating apparently robust circulating T cell responses, vaccines alone do not eradicate the brain tumors against which they are directed, nor do they provide gains in survival [81]. More encouragingly, however, when vaccines against brain tumors are used in conjunction with chemotherapy, the combination strategy has shown improvements in median progression-free and overall survival, although the emergence of antigen loss variants ultimately lead to tumor progression in a large majority of cases [82]. Thus, the promise of targeted vaccination strategies for treatment of CNS tumor patients remains an exciting area of research, but lacks sufficient efficacy to qualify as a standard therapy. For this reason, it is critical to understand the molecular mechanisms that contribute to tumor-related immune privilege in the CNS—especially those mechanisms that may be targeted by available therapeutic agents.

### 4.2. Pharmacological Blockade of Indoleamine 2,3-Dioxygenase

In various mouse tumor models, pharmacological inhibition of IDO can transiently break IDO-mediated tolerance and can improve the effectiveness of a number of chemotherapeutic agents, in an immune-mediated fashion [51, 52, 136]. A small molecule inhibitor of the IDO pathway (1-methyl-D-tryptophan, 1MT) is in Phase I and Phase II clinical trials for treatment of peripheral tumors in adult patients [145]. 1MT is not directly cytolytic to tumor cells [45, 52, 136, 145], but many chemotherapy agents are known to synergize with 1MT [52]. Recently, 1MT has been shown to reduce IDO activity in human-derived glioma cell preparations *in vitro* without diminishing the cytotoxicity of standard chemotherapeutic drugs, such as temozolomide [136]. However, no *in vivo* studies of 1MT have been reported, as yet, in preclinical brain-tumor models.

### 4.3. Antiangiogenesis Therapy

Despite the strong rationale behind developing antiangiogenic drug candidates [138], agents such as bevacizumab, a humanized monoclonal antibody that targets VEGF, have yielded mixed results [3, 146, 147]. Animal studies have shown anti-VEGF therapy to be effective at compromising glioblastoma perfusion by eliminating intratumoral vessels [146] via an apoptotic pathway [147]. However, intratumoral hypoxia appears to exert selection pressure upon glioma cells, increasing their invasive potential [146]. Furthermore, clinical trial data show conflicting results with significant extension of progression-free survival but no improvement in overall survival, relative to historical controls, in patients treated with bevacizumab and temozolomide [3]. These observations have raised the question of whether anti-angiogenic drugs may actually compromise delivery of adjuvant chemotherapy to the tumor bed and thereby decrease effective glioma drug exposure.

### 4.4. Other Potential Strategies for Breaking Tolerance to CNS Tumors

Other agents that may be beneficial for brain tumor immunotherapy are also approved or in the pipeline. Contact-mediated antagonism of T cell costimulation by ligation of CTLA-4 [11, 62] or PD-1 has been shown to inhibit tumor-directed T cell responses [11, 46, 62]. PD-L1, one of the ligands for PD-1, can be expressed by glioma cancer cells as a protective mechanism [66]. Recently, ipilimumab, a monoclonal antibody that blocks signaling through CTLA-4, was approved by the Food and Drug Administration (FDA) for use in treating metastatic melanoma [63–65], and it has begun Phase III clinical trials for use in metastatic castration-resistant prostate cancer [64]. In addition, a monoclonal antibody that targets PD-1 signaling is in early-phase clinical trials for solid tumors, including prostate cancer. Although these drugs have yet to be tested for efficacy in CNS tumors, they represent promising avenues of immunotherapy that may be useful in targeting brain tumor tolerance in the future.

## 5. Conclusions

Malignant central nervous system tumors are resistant to standard radiation and chemotherapy following surgical extirpation. The specialized immunology of the CNS excludes or attenuates effective immune responses in malignancies. However, despite the complexity of this “CNS immune privilege”, it is possible to recruit and activate lymphocytes and myeloid cells under certain conditions. Gaining a better understanding of CNS tumor-specific tolerogenic mechanisms is critically important to improving the survivability of this disease, which currently resists our most aggressive and multimodal therapeutic strategies.

 Tumor-induced immune tolerance is robust, because successful tumors have been selected throughout their existence for their ability to evade the immune system. Even during the earliest stages of tumorigenesis, when high cell turnover and availability of tumor shared “self” antigens have the potential to awaken the otherwise quiescent immune system, CNS tolerance mechanisms must be intact for tumor survival. The specialized stroma of CNS tumors is likely to be critical to maintenance of immune suppression within their “sterile inflammatory” microenvironment. Infiltrating microglia, macrophages, and astrocytes make up this stromal milieu and maintain tumor tolerance through a variety of mechanisms, including secretion of immune suppressive cytokines and growth factors, suppression of local T cell responses, and recruitment of regulatory T cells. Vaccination strategies to recruit the immune system to drive tumor clearance must first overcome these tolerogenic mechanisms. Promising new therapies, such as IDO-inhibitor drugs and other checkpoint-blockade strategies, used with vaccines in multimodal combination chemoimmunotherapy regimens, may allow immunologic therapy of brain tumors to reach its full potential.

## Figures and Tables

**Table 1:** Mechanisms of immune privilege.

General peripheral tolerance	Ref
T cell negative selection in thymus	[6]
Natural (thymic) Tregs	[7, 8]
Acquired (adaptive) Tregs	[9, 10]
Local immunosuppression (IDO, TGF-*β*, IL10, CTLA-4)	[11–27]

CNS-specific privilege	Ref

Reduced lymphatic transport to draining lymph nodes	[28–33]
Lack of resident immunogenic APCs (dendritic cells)	[28, 29, 34–37]
Specialized endothelium excludes naïve T cells	[28, 29, 34, 35, 38]
Local immunosuppression by astrocytes and microglia	[28, 35, 38–41]

Tumor-induced immunosuppression (CNS and non-CNS)	Ref

Local activation of natural Tregs	[7, 42–44]
Tumor-specific (adaptive) Tregs	[42, 44–48]
Local intratumoral immunosuppression	
IDO	[16, 45, 47–53]
Arginase	[42, 54, 55]
TGF-*β*	[56–59]
IL10	[60, 61]
CTLA-4	[11, 62–65]
PD-L1	[11, 46, 62, 66]
Myeloid-derived suppressor cells	[67–70]
Tolerogenic APCs	[42, 44, 45, 47, 48, 61, 71–73]
Tolerogenic draining lymph nodes	[45, 47, 48, 73]
Quiescent vascular endothelium	[74–79]

Tregs: regulatory T cells; IDO: indoleamine 2,3-dioxygenase; TGF-*β*: transforming growth factor-beta; IL10: interleukin-10; CTLA-4: cytotoxic T lymphocyte antigen-4; PD-L1: programmed death ligand-1; APCs: antigen-presenting cells.

## References

[1] Howlader N, Noone AM, Krapcho M (2011). *SEER Cancer Statistics Review 1975–2008*.

[2] * Section 28: Childhood Cancer by Site, 0–19 Year Old Age Group*.

[3] Lai A, Tran A, Nghiemphu PL (2011). Phase II study of bevacizumab plus temozolomide during and after radiation therapy for patients with newly diagnosed glioblastoma multiforme. *Journal of Clinical Oncology*.

[4] Lin AA, Tripathi PK, Sholl A, Jordan MB, Hildeman DA (2009). Gamma interferon signaling in macrophage lineage cells regulates central nervous system inflammation and chemokine production. *Journal of Virology*.

[5] Zhu X, Fallert-Junecko BA, Fujita M (2010). Poly-ICLC promotes the infiltration of effector T cells into intracranial gliomas via induction of CXCL10 in IFN-*α* and IFN-*γ* dependent manners. *Cancer Immunology, Immunotherapy*.

[6] Kishimoto H, Sprent J (1997). Negative selection in the thymus includes semimature T cells. *Journal of Experimental Medicine*.

[7] Miyara M, Sakaguchi S (2007). Natural regulatory T cells: mechanisms of suppression. *Trends in Molecular Medicine*.

[8] Jordan MS, Boesteanu A, Reed AJ (2001). Thymic selection of CD4^+^CD25^+^ regulatory T cells induced by an agonist self-peptide. *Nature Immunology*.

[9] Haribhai D, Williams JB, Jia S (2011). A requisite role for induced regulatory T cells in tolerance based on expanding antigen receptor diversity. *Immunity*.

[10] Thompson LJ, Valladao AC, Ziegler SF (2011). Cutting edge: de novo induction of functional Foxp3^+^ regulatory CD4 T cells in response to tissue-restricted self antigen. *Journal of Immunology*.

[11] Peggs KS, Quezada SA, Allison JP (2008). Cell intrinsic mechanisms of T-cell inhibition and application to cancer therapy. *Immunological Reviews*.

[12] von Boehmer H (2005). Mechanisms of suppression by suppressor T cells. *Nature Immunology*.

[13] Gorelik L, Flavell RA (2001). Immune-mediated eradication of tumors through the blockade of transforming growth factor-*β* signaling in T cells. *Nature Medicine*.

[14] Munn DH, Shafizadeh E, Attwood JT, Bondarev I, Pashine A, Mellor AL (1999). Inhibition of T cell proliferation by macrophage tryptophan catabolism. *Journal of Experimental Medicine*.

[15] Attwood JT, Munn DH (1999). Macrophage suppression of T cell activation: a potential mechanism of peripheral tolerance. *International Reviews of Immunology*.

[16] Munn DH, Sharma MD, Lee JR (2002). Potential regulatory function of human dendritic cells expressing indoleamine 2,3-dioxygenase. *Science*.

[17] Munn DH, Zhou M, Attwood JT (1998). Prevention of allogeneic fetal rejection by tryptophan catabolism. *Science*.

[18] Mellor AL, Keskin DB, Johnson T, Chandler P, Munn DH (2002). Cells expressing indoleamine 2,3-dioxygenase inhibit T cell responses. *Journal of Immunology*.

[19] Baban B, Chandler P, McCool D, Marshall B, Munn DH, Mellor AL (2004). Indoleamine 2,3-dioxygenase expression is restricted to fetal trophoblast giant cells during murine gestation and is maternal genome specific. *Journal of Reproductive Immunology*.

[20] Baban B, Chandler PR, Sharma MD (2009). IDO activates regulatory T cells and blocks their conversion into Th17-like T cells. *Journal of Immunology*.

[21] Baban B, Hansen AM, Chandler PR (2005). A minor population of splenic dendritic cells expressing CD19 mediates IDO-dependent T cell suppression via type I IFN signaling following B7 ligation. *International Immunology*.

[22] Mellor AL, Chandler P, Baban B (2004). Specific subsets of murine dendritic cells acquire potent T cell regulatory functions following CTLA4-mediated induction of indoleamine 2,3 dioxygenase. *International Immunology*.

[23] Fallarino F, Vacca C, Orabona C (2002). Functional expression of indoleamine 2,3-dioxygenase by murine CD8*α*+ dendritic cells. *International Immunology*.

[24] Grohmann U, Volpi C, Fallarino F (2007). Reverse signaling through GITR ligand enables dexamethasone to activate IDO in allergy. *Nature Medicine*.

[25] Coquerelle C, Oldenhove G, Acolty V (2009). Anti-CTLA-4 treatment induces IL-10-producing ICOS^+^ regulatory T cells displaying IDO-dependent anti-inflammatory properties in a mouse model of colitis. *Gut*.

[26] Raftery MJ, Wieland D, Gronewald S, Kraus AA, Giese T, Schönrich G (2004). Shaping phenotype, function, and survival of dendritic cells by cytomegalovirus-encoded IL-10. *Journal of Immunology*.

[27] Gorelik L, Flavell RA (2002). Transforming growth factor-*β* in T-cell biology. *Nature Reviews Immunology*.

[28] Mitchell DA, Fecci PE, Sampson JH (2008). Immunotherapy of malignant brain tumors. *Immunological Reviews*.

[29] Engelhardt B, Ransohoff RM (2005). The ins and outs of T-lymphocyte trafficking to the CNS: anatomical sites and molecular mechanisms. *Trends in Immunology*.

[30] Hatterer E, Davoust N, Didier-Bazes M (2006). How to drain without lymphatics? Dendritic cells migrate from the cerebrospinal fluid to the B-cell follicles of cervical lymph nodes. *Blood*.

[31] Weller RO, Galea I, Carare RO, Minagar A (2010). Pathophysiology of the lymphatic drainage of the central nervous system: implications for pathogenesis and therapy of multiple sclerosis. *Pathophysiology*.

[32] Hochmeister S, Zeitelhofer M, Bauer J (2008). After injection into the striatum, in vitro-differentiated microglia- and bone marrow-derived dendritic cells can leave the central nervous system via the blood stream. *The American Journal of Pathology*.

[33] Harling-Berg CJ, Park JT, Knopf PM (1999). Role of the cervical lymphatics in the Th2-type hierarchy of CNS immune regulation. *Journal of Neuroimmunology*.

[34] Rezai-Zadeh K, Gate D, Town T (2009). CNS infiltration of peripheral immune cells: D-Day for neurodegenerative disease?. *Journal of Neuroimmune Pharmacology*.

[35] Fabry Z, Schreiber HA, Harris MG, Sandor M (2008). Sensing the microenvironment of the central nervous system: immune cells in the central nervous system and their pharmacological manipulation. *Current Opinion in Pharmacology*.

[36] Nimmerjahn A, Kirchhoff F, Helmchen F (2005). Neuroscience: resting microglial cells are highly dynamic surveillants of brain parenchyma in vivo. *Science*.

[37] Guillemin GJ, Brew BJ (2004). Microglia, macrophages, perivascular macrophages, and pericytes: a review of function and identification. *Journal of Leukocyte Biology*.

[38] Mrass P, Weninger W (2006). Immune cell migration as a means to control immune privilege: lessons from the CNS and tumors. *Immunological Reviews*.

[39] Kaur G, Han SJ, Yang I, Crane C (2010). Microglia and central nervous system immunity. *Neurosurgery Clinics of North America*.

[40] Gimsa U, Øren A, Pandiyan P (2004). Astrocytes protect the CNS: antigen-specific T helper cell responses are inhibited by astrocyte-induced upregulation of CTLA-4 (CD152). *Journal of Molecular Medicine*.

[41] Kwidzinski E, Bunse J, Kovac AD (2003). IDO (indolamine 2,3-dioxygenase) expression and function in the CNS. *Advances in Experimental Medicine and Biology*.

[42] Mellor AL, Munn DH (2008). Creating immune privilege: active local suppression that benefits friends, but protects foes. *Nature Reviews Immunology*.

[43] Darrasse-Jèze G, Bergot AS, Durgeau A (2009). Tumor emergence is sensed by self-specific CD44^hi^ memory Tregs that create a dominant tolerogenic environment for tumors in mice. *Journal of Clinical Investigation*.

[44] Munn DH (2011). Indoleamine 2,3-dioxygenase, Tregs and cancer. *Current Medicinal Chemistry*.

[45] Sharma MD, Hou DY, Baban B (2010). Reprogrammed Foxp3^+^ Regulatory T Cells Provide Essential Help to Support Cross-presentation and CD8^+^ T Cell Priming in Naive Mice. *Immunity*.

[46] Francisco LM, Salinas VH, Brown KE (2009). PD-L1 regulates the development, maintenance, and function of induced regulatory T cells. *Journal of Experimental Medicine*.

[47] Sharma MD, Baban B, Chandler P (2007). Plasmacytoid dendritic cells from mouse tumor-draining lymph nodes directly activate mature Tregs via indoleamine 2,3-dioxygenase. *Journal of Clinical Investigation*.

[48] Sharma MD, Hou DY, Liu Y (2009). Indoleamine 2,3-dioxygenase controls conversion of Foxp3^+^ Tregs to TH17-like cells in tumor-draining lymph nodes. *Blood*.

[49] Muller AJ, Sharma MD, Chandler PR (2008). Chronic inflammation that facilitates tumor progression creates local immune suppression by inducing indoleamine 2,3-dioxygenase. *Proceedings of the National Academy of Sciences of the United States of America*.

[50] Uyttenhove C, Pilotte L, Théate I (2003). Evidence for a tumoral immune resistance mechanism based on tryptophan degradation by indoleamine 2,3-dioxygenase. *Nature Medicine*.

[51] Hou DY, Muller AJ, Sharma MD (2007). Inhibition of indoleamine 2,3-dioxygenase in dendritic cells by stereoisomers of 1-methyl-tryptophan correlates with antitumor responses. *Cancer Research*.

[52] Muller AJ, DuHadaway JB, Donover PS, Sutanto-Ward E, Prendergast GC (2005). Inhibition of indoleamine 2,3-dioxygenase, an immunoregulatory target of the cancer suppression gene Bin1, potentiates cancer chemotherapy. *Nature Medicine*.

[53] Muller AJ, Duhadaway JB, Jaller D, Curtis P, Metz R, Prendergast GC (2010). Immunotherapeutic suppression of indoleamine 2,3-dioxygenase and tumor growth with ethyl pyruvate. *Cancer Research*.

[54] Sharda DR, Yu S, Ray M (2011). Regulation of macrophage arginase expression and tumor growth by the Ron receptor tyrosine kinase. *Journal of Immunology*.

[55] Singer K, Gottfried E, Kreutz M, Mackensen A (2011). Suppression of T-cell responses by tumor metabolites. *Cancer Immunology, Immunotherapy*.

[56] Platten M, Wick W, Weller M (2001). Malignant glioma biology: role for TGF-*β* in growth, motility, angiogenesis, and immune escape. *Microscopy Research and Technique*.

[57] Wesolowska A, Kwiatkowska A, Slomnicki L (2008). Microglia-derived TGF-*β* as an important regulator of glioblastoma invasion—an inhibition of TGF-*β*-dependent effects by shRNA against human TGF-*β* type II receptor. *Oncogene*.

[58] Wick W, Platten M, Weller M (2001). Glioma cell invasion: regulation of metalloproteinase activity by TGF-*β*. *Journal of Neuro-Oncology*.

[59] Flavell RA, Sanjabi S, Wrzesinski SH, Licona-Limón P (2010). The polarization of immune cells in the tumour environment by TGFÎ 2. *Nature Reviews Immunology*.

[60] Huettner C, Czub S, Kerkau S, Roggendorf W, Tonn JC (1997). Interleukin 10 is expressed in human gliomas in vivo and increases glioma cell proliferation and motility in vitro. *Anticancer Research*.

[61] Wu A, Wei J, Kong LY (2010). Glioma cancer stem cells induce immunosuppressive macrophages/microglia. *Neuro-Oncology*.

[62] Schreiber RD, Old LJ, Smyth MJ (2011). Cancer immunoediting: integrating immunity's roles in cancer suppression and promotion. *Science*.

[63] Cameron F, Whiteside G, Perry C (2011). Ipilimumab: first global approval. *Drugs*.

[64] May KF, Gulley JL, Drake CG, Dranoff G, Kantoff PW (2011). Prostate cancer immunotherapy. *Clinical Cancer Research*.

[65] Robert C, Thomas L, Bondarenko I (2011). Ipilimumab plus dacarbazine for previously untreated metastatic melanoma. *The New England Journal of Medicine*.

[66] Parsa AT, Waldron JS, Panner A (2007). Loss of tumor suppressor PTEN function increases B7-H1 expression and immunoresistance in glioma. *Nature Medicine*.

[67] Murdoch C, Muthana M, Coffelt SB, Lewis CE (2008). The role of myeloid cells in the promotion of tumour angiogenesis. *Nature Reviews Cancer*.

[68] Qian BZ, Pollard JW (2010). Macrophage diversity enhances tumor progression and metastasis. *Cell*.

[69] Ribechini E, Greifenberg V, Sandwick S, Lutz MB (2010). Subsets, expansion and activation of myeloid-derived suppressor cells. *Medical Microbiology & Immunology*.

[70] Lechner MG, Liebertz DJ, Epstein AL (2010). Characterization of cytokine-induced myeloid-derived suppressor cells from normal human peripheral blood mononuclear cells. *Journal of Immunology*.

[71] Biswas SK, Mantovani A (2010). Macrophage plasticity and interaction with lymphocyte subsets: cancer as a paradigm. *Nature Immunology*.

[72] Yang I, Han SJ, Kaur G, Crane C, Parsa AT (2010). The role of microglia in central nervous system immunity and glioma immunology. *Journal of Clinical Neuroscience*.

[73] Ma Y, Aymeric L, Locher C, Kroemer G, Zitvogel L (2011). The dendritic cell-tumor cross-talk in cancer. *Current Opinion in Immunology*.

[74] Sunderkotter C, Steinbrink K, Goebeler M, Bhardwaj R, Sorg C (1994). Macrophages and angiogenesis. *Journal of Leukocyte Biology*.

[75] Lund AW, Swartz MA (2010). Role of lymphatic vessels in tumor immunity: passive conduits or active participants?. *Journal of Mammary Gland Biology and Neoplasia*.

[76] Wu NZ, Klitzman B, Dodge R, Dewhirst MW (1992). Diminished leukocyte-endothelium interaction in tumor microvessels. *Cancer Research*.

[77] Zadeh G, Koushan K, Pillo L, Shannon P, Guha A (2004). Role of Ang1 and its interaction with VEGF-A in astrocytomas. *Journal of Neuropathology and Experimental Neurology*.

[78] Brunckhorst MK, Wang H, Lu R, Yu Q (2010). Angiopoietin-4 promotes glioblastoma progression by enhancing tumor cell viability and angiogenesis. *Cancer Research*.

[79] Debinski W, Slagle-Webb B, Achen MG (2001). VEGF-D is an X-linked/AP-1 regulated putative onco-angiogen in human glioblastoma multiforme. *Molecular Medicine*.

[80] Terry LA, Usherwood EJ, Lees S, Macintyre N, Nash AA (1997). Immune response to murine cell lines of glial origin transplanted into the central nervous system of adult mice. *Immunology*.

[81] Liau LM, Prins RM, Kiertscher SM (2005). Dendritic cell vaccination in glioblastoma patients induces systemic and intracranial T-cell responses modulated by the local central nervous system tumor microenvironment. *Clinical Cancer Research*.

[82] Sampson JH, Aldape KD, Archer GE (2011). Greater chemotherapy-induced lymphopenia enhances tumor-specific immune responses that eliminate EGFRvIII-expressing tumor cells in patients with glioblastoma. *Neuro-Oncology*.

[83] Zhai H, Heppner FL, Tsirka SE (2011). Microglia/macrophages promote glioma progression. *GLIA*.

[84] Engelhardt B (2008). Immune cell entry into the central nervous system: involvement of adhesion molecules and chemokines. *Journal of the Neurological Sciences*.

[85] Engelhardt B, Wolburg H (2004). Mini review: transendothelial migration of leukocytes: through the front door or around the side of the house?. *European Journal of Immunology*.

[86] Medawar PB (1948). Immunity to homologous grafted skin; the fate of skin homografts. *British Journal of Experimental Pathology*.

[87] Kraman M, Bambrough PJ, Arnold JN (2010). Suppression of antitumor immunity by stromal cells expressing fibroblast activation protein-*α*. *Science*.

[88] Shields JD, Kourtis IC, Tomei AA, Roberts JM, Swartz MA (2010). Induction of lymphoidlike stroma and immune escape by tumors that express the chemokine CCL21. *Science*.

[89] Kubota R, Yamada S, Kubota K, Ishiwata K, Tamahashi N, Ido T (1992). Intratumoral distribution of fluorine-18-fluorodeoxyglucose in vivo: high accumulation in macrophages and granulation tissues studied by microautoradiography. *Journal of Nuclear Medicine*.

[90] Biegel JA (1999). Cytogenetics and molecular genetics of childhood brain tumors. *Neuro-Oncology*.

[91] Sanson M, Thillet J, Hoang-Xuan K (2004). Molecular changes in gliomas. *Current Opinion in Oncology*.

[92] Speidel D (2010). Transcription-independent p53 apoptosis: an alternative route to death. *Trends in Cell Biology*.

[93] Wechsler-Reya R, Scott MP (2001). The developmental biology of brain tumors. *Annual Review of Neuroscience*.

[94] Llambi F, Green DR (2011). Apoptosis and oncogenesis: give and take in the BCL-2 family. *Current Opinion in Genetics and Development*.

[95] Zhou F, Yang Y, Xing D (2011). Bcl-2 and Bcl-xL play important roles in the crosstalk between autophagy and apoptosis. *FEBS Journal*.

[96] Mills JR, Hippo Y, Robert F (2008). mTORC1 promotes survival through translational control of Mcl-1. *Proceedings of the National Academy of Sciences of the United States of America*.

[97] Schietinger A, Philip M, Schreiber H (2008). Specificity in cancer immunotherapy. *Seminars in Immunology*.

[98] Duan F, Lin Y, Liu C (2009). Immune rejection of mouse tumors expressing Mutated self. *Cancer Research*.

[99] Mantovani A, Allavena P, Sica A, Balkwill F (2008). Cancer-related inflammation. *Nature*.

[100] Movahedi K, Laoui D, Gysemans C (2010). Different tumor microenvironments contain functionally distinct subsets of macrophages derived from Ly6C(high) monocytes. *Cancer Research*.

[101] Clark CE, Beatty GL, Vonderheide RH (2009). Immunosurveillance of pancreatic adenocarcinoma: insights from genetically engineered mouse models of cancer. *Cancer Letters*.

[102] Willimsky G, Blankenstein T (2005). Sporadic immunogenic tumours avoid destruction by inducing T-cell tolerance. *Nature*.

[103] Zhang B, Bowerman NA, Salama JK (2007). Induced sensitization of tumor stroma leads to eradication of established cancer by T cells. *Journal of Experimental Medicine*.

[104] Quezada SA, Peggs KS, Simpson TR, Allison JP (2011). Shifting the equilibrium in cancer immunoediting: from tumor tolerance to eradication. *Immunological Reviews*.

[105] Quezada SA, Peggs KS, Simpson TR, Shen Y, Littman DR, Allison JP (2008). Limited tumor infiltration by activated T effector cells restricts the therapeutic activity of regulatory T cell depletion against established melanoma. *Journal of Experimental Medicine*.

[106] Schreiber H, Rowley DA (2010). Awakening immunity. *Science*.

[107] Dunn JR, Reed JE, Du Plessis DG (2006). Expression of ADAMTS-8, a secreted protease with antiangiogenic properties, is downregulated in brain tumours. *British Journal of Cancer*.

[108] Fletcher AL, Malhotra D, Turley SJ (2011). Lymph node stroma broaden the peripheral tolerance paradigm. *Trends in Immunology*.

[109] He Z, Bateman A (2003). Progranulin (granulin-epithelin precursor, PC-cell-derived growth factor, acrogranin) mediates tissue repair and tumorigenesis. *Journal of Molecular Medicine*.

[110] Liau LM, Lallone RL, Seitz RS (2000). Identification of a human glioma-associated growth factor gene, granulin, using differential immuno-absorption. *Cancer Research*.

[111] Han SJ, Kaur G, Yang I, Lim M (2010). Biologic principles of immunotherapy for malignant gliomas. *Neurosurgery Clinics of North America*.

[112] Wermeling F, Karlsson MCI, McGaha TL (2009). An anatomical view on macrophages in tolerance. *Autoimmunity Reviews*.

[113] Asano K, Nabeyama A, Miyake Y (2010). CD169-positive macrophages dominate antitumor immunity by crosspresenting dead cell-associated antigens. *Immunity*.

[114] Giometto B, Bozza F, Faresin F, Alessio L, Mingrino S, Tavolato B (1996). Immune infiltrates and cytokines in gliomas. *Acta Neurochirurgica*.

[115] Merzak A, McCrea S, Koocheckpour S, Pilkington GJ (1994). Control of human glioma cell growth, migration and invasion in vitro by transforming growth factor *β*1. *British Journal of Cancer*.

[116] Zindl CL, Chaplin DD (2010). Tumor immune evasion. *Science*.

[117] Qualls JE, Murray PJ (2010). A double agent in cancer: stopping macrophages wounds tumors. *Nature Medicine*.

[118] Kluwe J, Mencin A, Schwabe RF (2009). Toll-like receptors, wound healing, and carcinogenesis. *Journal of Molecular Medicine*.

[119] Restifo NP (2010). Can antitumor immunity help to explain “oncogene addiction”?. *Cancer Cell*.

[120] Tseng D, Vasquez-Medrano DA, Brown JM (2011). Targeting SDF-1/CXCR4 to inhibit tumour vasculature for treatment of glioblastomas. *British Journal of Cancer*.

[121] Frumento G, Piazza T, Di Carlo E, Ferrini S (2006). Targeting tumor-related immunosuppression for cancer immunotherapy. *Endocrine, Metabolic and Immune Disorders—Drug Targets*.

[122] Green DR, Ferguson T, Zitvogel L, Kroemer G (2009). Immunogenic and tolerogenic cell death. *Nature Reviews Immunology*.

[123] de Visser KE, Eichten A, Coussens LM (2006). Paradoxical roles of the immune system during cancer development. *Nature Reviews Cancer*.

[124] Paupert J, Sounni NE, Noël A (2011). Lymphangiogenesis in post-natal tissue remodeling: lymphatic endothelial cell connection with its environment. *Molecular Aspects of Medicine*.

[125] Trajkovic V, Vuckovic O, Stosic-Grujicic S (2004). Astrocyte-induced regulatory T cells mitigate CNS autoimmunity. *GLIA*.

[126] Mellor AL, Munn DH (2011). Physiologic control of the functional status of Foxp3^+^ regulatory T cells. *Journal of Immunology*.

[127] Munn DH, Sharma MD, Hou D (2004). Expression of indoleamine 2,3-dioxygenase by plasmacytoid dendritic cells in tumor-draining lymph nodes. *Journal of Clinical Investigation*.

[128] Munn DH, Sharma MD, Mellor AL (2004). Ligation of B7-1/B7-2 by human CD4^+^ T cells triggers indoleamine 2,3-dioxygenase activity in dendritic cells. *Journal of Immunology*.

[129] Yan ML, Wang YD, Tian YF, Lai ZD, Yan LN (2010). Inhibition of allogeneic T-cell response by Kupffer cells expressing indoleamine 2,3-dioxygenase. *World Journal of Gastroenterology*.

[130] Soliman H, Mediavilla-Varela M, Antonia S (2010). Indoleamine 2,3-dioxygenase is it an immune suppressor?. *Cancer Journal*.

[131] Yan Y, Zhang GX, Gran B (2010). IDO upregulates regulatory T cells via tryptophan catabolite and suppresses encephalitogenic T cell responses in Experimental autoimmune encephalomyelitis. *Journal of Immunology*.

[132] Munn DH, Sharma MD, Baban B (2005). GCN2 kinase in T cells mediates proliferative arrest and anergy induction in response to indoleamine 2,3-dioxygenase. *Immunity*.

[133] Mellor AL, Munn D, Chandler P (2003). Tryptophan catabolism and T cell responses. *Advances in Experimental Medicine and Biology*.

[134] Sun Y, Chin YE, Weisiger E (2009). Cutting edge: negative regulation of dendritic cells through acetylation of the nonhistone protein STAT-3. *Journal of Immunology*.

[135] Bonifazi P, Zelante T, D’Angelo C (2009). Balancing inflammation and tolerance in vivo through dendritic cells by the commensal Candida albicans. *Mucosal Immunology*.

[136] Miyazaki T, Moritake K, Yamada K (2009). Indoleamine 2,3-dioxygenase as a new target for malignant glioma therapy: laboratory investigation. *Journal of Neurosurgery*.

[137] Moffett JR, Els T, Espey MG, Walter SA, Streit WJ, Namboodiri MAA (1997). Quinolinate immunoreactivity in experimental rat brain tumors is present in macrophages but not in astrocytes. *Experimental Neurology*.

[138] Chen Y, Jing Z, Luo C Vasculogenic mimicry-potential target for glioblastoma therapy: an in vitro and in vivo study.

[139] Ricci-Vitiani L, Pallini R, Biffoni M (2010). Tumour vascularization via endothelial differentiation of glioblastoma stem-like cells. *Nature*.

[140] Shaifer CA, Huang J, Lin PC (2010). Glioblastoma cells incorporate into tumor vasculature and contribute to vascular radioresistance. *International Journal of Cancer*.

[141] Virrey JJ, Golden EB, Sivakumar W (2009). Glioma-associated endothelial cells are chemoresistant to temozolomide. *Journal of Neuro-Oncology*.

[142] Wang R, Chadalavada K, Wilshire J (2010). Glioblastoma stem-like cells give rise to tumour endothelium. *Nature*.

[143] El Hallani S, Boisselier B, Peglion F (2010). A new alternative mechanism in glioblastoma vascularization: tubular vasculogenic mimicry. *Brain*.

[144] Prins RM, Shu CJ, Radu CG (2008). Anti-tumor activity and trafficking of self, tumor-specific T cells against tumors located in the brain. *Cancer Immunology, Immunotherapy*.

[145] Cheever MA (2008). Twelve immunotherapy drugs that could cure cancers. *Immunological Reviews*.

[146] Keunen O, Johansson M, Oudin A (2011). Anti-VEGF treatment reduces blood supply and increases tumor cell invasion in glioblastoma. *Proceedings of the National Academy of Sciences of the United States of America*.

[147] Naik E, O'Reilly LA, Asselin-Labat M-L (2011). Destruction of tumor vasculature and abated tumor growth upon VEGF blockade is driven by proapoptotic protein Bim in endothelial cells. *Journal of Experimental Medicine*.

